# *Mycobacterium smegmatis* enhances shikonin-induced immunogenic cell death—an efficient *in situ* tumor vaccine strategy

**DOI:** 10.7555/JBR.38.20240049

**Published:** 2024-05-29

**Authors:** Zhaoye Qian, Zhe Zhang, Lanqi Cen, Yaohua Ke, Jie Shao, Manman Tian, Baorui Liu

**Affiliations:** 1 Department of Oncology, Nanjing Drum Tower Hospital Clinical College of Nanjing Medical University, Nanjing, Jiangsu 210008, China; 2 Department of Oncology, the Affiliated Huai'an No. 1 People's Hospital, Nanjing Medical University, Huai'an, Jiangsu 223000, China; 3 Center of Clinical Oncology, the Affiliated Hospital of Xuzhou Medical University, Xuzhou, Jiangsu 221000, China; 4 Department of Oncology, China Pharmaceutical University Nanjing Drum Tower Hospital, Nanjing, Jiangsu 210008, China; 5 The Comprehensive Cancer Center, Nanjing Drum Tower Hospital, Affiliated Hospital of Medical School, Nanjing University, Nanjing, Jiangsu 210008, China; 6 Department of Oncology, Nanjing Drum Tower Hospital, Affiliated Hospital of Medical School, Nanjing University, Nanjing, Jiangsu 210008, China

**Keywords:** *Mycobacterium smegmatis*, shikonin, immunogenic cell death, tumor vaccines, immunogenicity, cytotoxicity

## Abstract

Tumor vaccines are a promising avenue in cancer immunotherapy. Despite the progress in targeting specific immune epitopes, tumor cells lacking these epitopes can evade the treatment. Here, we aimed to construct an efficient *in situ* tumor vaccine called Vac-SM, utilizing shikonin (SKN) to induce immunogenic cell death (ICD) and *Mycobacterium smegmatis* as an immune adjuvant to enhance *in situ* tumor vaccine efficacy. SKN showed a dose-dependent and time-dependent cytotoxic effect on the tumor cell line and induced ICD in tumor cells as evidenced by the CCK-8 assay and the detection of the expression of relevant indicators, respectively. Compared with the control group, the *in situ* Vac-SM injection in mouse subcutaneous metastatic tumors significantly inhibited tumor growth and distant tumor metastasis, while also improving survival rates. *Mycobacterium* *smegmatis* effectively induced maturation and activation of bone marrow-derived dendritic cells (DCs), and *in vivo* tumor-draining lymph nodes showed an increased maturation of DCs and a higher proportion of effector memory T-cell subsets with the Vac-SM treatment, based on flow cytometry analysis results. Collectively, the Vac-SM vaccine effectively induces ICD, improves antigen presentation by DCs, activates a specific systemic antitumor T-cell immune response, exhibits a favorable safety profile, and holds the promise for clinical translation for local tumor immunotherapy.

## Introduction

Tumor vaccines are a promising approach to cancer immunotherapy^[1]^. Using high-throughput sequencing technology, personalized antigen vaccines have recently been rapidly developed^[2]^. These "predefined antigen" vaccines, use immune epitopes formed by tumor mutations, identified by high-throughput sequencing. They are designed to target one or several sites to generate a strong immunogenicity^[3]^. However, they also have limitations. Tumors with the high heterogeneity often evade treatments, because certain tumor cells do not express the immune epitopes and thus evade immune killing. Therefore, *in situ* tumor vaccines present a promising form of vaccination^[4]^. Unlike the singular and strong immunogenicity observed in "predefined antigen" vaccines, the *in situ* tumor vaccines induce immunogenic cell death (ICD) within the tumor site. This process releases abundant damage-associated molecular patterns (DAMPs), resulting in a comprehensive and diverse collection of antigens characterized by potent immunogenicity^[1,5]^. Because of the local immunosuppressive microenvironment within tumors and the absence of antigen-presenting cells (APCs), inducing ICD alone proves to be inadequate for generating a strong and lasting antitumor immune response. Moreover, tumor antigens on the cell membrane are unable to directly bind to the major histocompatibility complex and must be internalized and processed by dendritic cells (DCs) for antigen presentation. Therefore, the *in situ* vaccines frequently require adjuvants to activate DCs and ensure effective antigen presentation^[6]^.

ICD is a distinctive form of cell death that triggers the immune system to identify and eliminate tumor cells^[7]^. During ICD, dying cells release various immune-stimulating molecules, including high-mobility group box 1 (HMGB1), adenosine triphosphate (ATP), and reactive oxygen species (ROS), acting as "eat me" signals. These molecules attract and activate DCs, facilitating antigen presentation and T-cell activation, as well as enhancing tumor-specific immune responses^[8]^. Therefore, inducers of ICD are crucial in tumor treatment to serve as *in situ* tumor vaccines. By inducing ICD in tumor cells, these agents transform the tumor site into an immune activation site, prompting the immune system to identify and target the tumor, thereby enhancing the overall immune response against the tumor^[9]^.

Shikonin (SKN), the principal component of *Lithospermum*, and its derivatives, including β, β′-dimethylacrylshikonin and isobutyrylshikonin, have shown various pharmacological effects, including anti-inflammatory, antibacterial, analgesic, cardiovascular protection, anti-obesity, and neuroprotective properties^[10–14]^. Moreover, SKN has shown a considerable potential with regard to antitumor activity. SKN, in synergy with traditional chemotherapeutic drugs, effectively inhibits the growth of liver cancer and enhances the efficacy of specific drugs, while reducing drug resistance^[15–16]^. Furthermore, SKN is a potent inducer of ICD, enhancing cellular oxidative stress by activating mitochondrion- and receptor-mediated apoptosis pathways, thereby promoting the effectiveness of DC-based immunotherapy^[17–18]^. Hence, SKN is extensively used in tumor immunotherapy, particularly as an adjuvant for DC vaccines.

Bacteria-derived components, including inactivated or attenuated bacteria, lipids, proteins, and nucleic acids, can activate innate immune responses. This mobilizes the immune system in response to foreign danger signals^[19]^. *Mycobacterium smegmatis* (*M. smegmatis*) exhibits a significant anticancer potential in immunotherapy, specifically in bladder cancer^[20–21]^. This bacterium directly affects cancer cells by inhibiting their proliferation but inducing apoptosis and activating immune cells, including macrophages, thereby strengthening the immune response. Its immunostimulatory functions are predominantly attributed to its cell wall components. *M. smegmatis* provokes a systemic immune response mediated by CD8^+^ T cells in a subcutaneous thymoma model and activates novel inflammatory DCs in the lymph nodes^[22]^. Moreover, *M. smegmatis* exhibits a rapid growth, is non-pathogenic, and has minimal side effects, making it a promising tool in tumor immunotherapy. Notably, it only induces diseases in a small fraction of individuals with compromised immune function.

**Figure 1:**
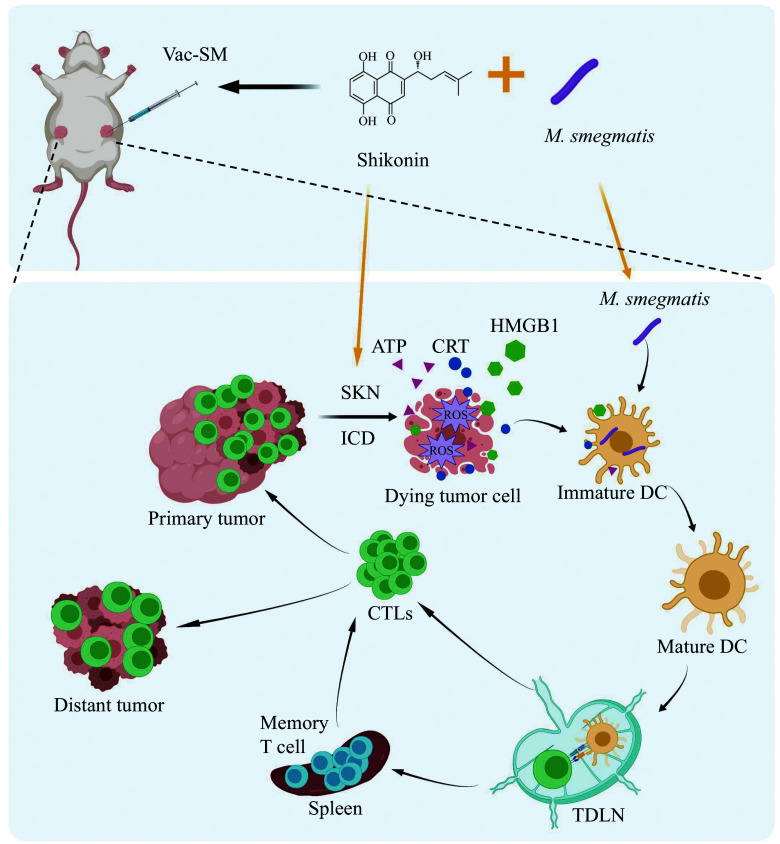
Schematic representation of the bifunctional *in situ* tumor vaccine named Vac-SM, merging the traditional Chinese herbal component SKN with the inactivated bacterial adjuvant (*Mycobacterium smegmatis*).

In the current study, we formulated a dual-function *in situ* tumor vaccine named Vac-SM for intratumoral injection to control tumor growth, merging the traditional Chinese herbal component SKN with the inactivated bacterial adjuvant *M. smegmatis* (***Fig. 1***).

## Materials and methods

### Cell lines, bacteria, and animals

The CT26 (mouse colon cancer cell) cell line was purchased from the Cell Bank of the Chinese Academy of Sciences (Beijing, China). The CT26 cells were cultured in RPMI-1640 complete medium (containing 10% FBS, 100 U/mL penicillin, and 100 μg/mL streptomycin) in an incubator at 37 ℃ with 5% CO_2_ and saturated humidity. The *M. smegmatis* (mc^2^155) strain was preserved at the Oncology Laboratory of Nanjing Drum Tower Hospital. Female BALB/c mice aged 4–6 weeks were acquired from Shanghai Sippr-BK Lab Animal Co., Ltd. (Shanghai, China). All mice were housed in the specific pathogen-free-grade Experimental Animal Center of the Cancer Research Institute of Nanjing Drum Tower Hospital. All animal studies, including euthanasia, were conducted following the guidelines and regulations of the Animal Protection Association of Medical Institutions at Nanjing Drum Tower Hospital, following the AAALAC and IACUC guidelines.

### ICD-related biochemical analysis

A fresh cell culture medium was introduced 12 h after the CT26 cells were plated, and varying concentrations of SKN were added based on the experimental groups. Following different culture periods, the cells were harvested for subsequent analysis. The ROS contents in the cells were measured by using a ROS assay kit (Beyotime, Shanghai, China). After incubating the cells with the DCFH-DA probe for 20 min, fluorescence was detected using a full-wavelength microplate reader (with excitation and emission wavelengths at 488 nm and 525 nm, respectively). The relative fluorescence intensity for each group was determined by dividing the fluorescence values by that of the control group (0 μg/mL). The ATP content of the cells was assessed using an ATP assay kit (Beyotime). Cells were collected and lysed using the lysis buffer provided in the kit. Then, 20 μL of each sample and various concentrations of standards were added to a 96-well plate preloaded with a luciferase working solution. Chemiluminescence was assessed using a full-wavelength microplate reader, and the ATP concentration was calculated based on the standard curve.

### Western blotting analysis

Western blotting was performed for HMGB1 and calreticulin (CRT) detection. Following cell harvest, RIPA lysis buffer (#P0013C, Beyotime) was used for cell lysis. Subsequently, the samples were prepared by adding a loading buffer for SDS-PAGE. The protein bands were then transferred onto membranes. The membranes were blocked and incubated with primary antibodies targeting HMGB1 (1∶1000; Cat. #AF1174, Beyotime) and CRT (1∶1000; Cat. #27298-1-AP, Beyotime), respectively, with GAPDH (1∶10000; Cat. #AF1186, Beyotime) as an internal reference. After incubation with secondary antibodies, the bands were scanned and photographed using an automatic chemiluminescence analyzer. The gray values were analyzed using ImageJ.

### Bone marrow-derived dendritic cell (BMDC) stimulation *in vitro*

To obtain BMDCs, healthy BALB/c mice (10-week-old, female) were anesthetized using isoflurane inhalation and euthanized by cervical dislocation. The femurs and tibias were aseptically dissected, and the ends of the long bones were cut to flush out bone marrow cells with saline. The cell suspension was subsequently filtered through a mesh to obtain a single-cell suspension. Red blood cells were lysed, and the remaining cells were seeded in a 6-well plate with RPMI-1640 medium with GM-CSF (20 ng/mL) and IL-4 (10 ng/mL). Half of the medium was replaced every 2 days with the removal of floating cells. On day 9, the culture medium was changed to serum- and cytokine-free media. The cells were stimulated with various agents for 24 h and then collected for flow cytometry analysis.

### Animal experiment

CT-26 tumor cells were cultured until they reached 80%–90% confluence, followed by trypsinization and cell collection. The cells were then prepared at a concentration of 5 × 10^7^ cells in 100 μL of PBS. Each mouse received a subcutaneous injection of the cell suspension in the left inguinal region. For the bilateral tumor models, an additional inoculation was performed in the right inguinal region of the same mouse. Tumor dimensions, including length (a) and width (b), were measured using a caliper. On day 7, when the tumor volume reached approximately 50 mm^3^, intratumoral injections were administered to treat the left-sided tumor in the unilateral tumor model. The same procedure was applied to the bilateral tumor model.

### Flow cytometry

Samples from the tumor tissue, spleen, and tumor-draining lymph nodes (TDLNs) were collected for analysis seven days after the last treatment. After anatomical dissection, the samples were washed and minced for tumor tissue sample preparation, followed by digestion with type Ⅳ collagenase for 2 to 4 h with stirring every 30 min. The samples were then filtered and centrifuged to obtain single-cell suspensions in PBS. Red blood cells were lysed, and the suspension was set aside. For the spleen and lymph node tissues, a simple grinding method was employed to disrupt the tissue structure, followed by filtration and red blood cell lysis to prepare a single-cell suspension. Before staining, the cells were washed three times with PBS. Antibodies were introduced into the samples and incubated at 4 ℃ in the dark to prevent exposure to light for 30 min. Excess antibodies were washed off before analysis. FITC-CD11c (Cat. #117305, Biolegend, San Diego, CA, USA), APC-CD80 (Cat. #104714, Biolegend), and PE-CD86 (Cat. #105008, Biolegend) were employed to detect DCs, and the CD11c^+^ CD80^+^CD86^+^ cell population represented mature DCs (mDCs). FITC-CD3 (Cat. #152304, Biolegend), Percp-Cy5.5-CD8 (Cat. #100733, Biolegend), PE-Cy7-CD62L (Cat. #104418, Biolegend), and PE-CD44 (Cat. #103008, Biolegend) were used for T-cell analysis, with the CD3^+^CD8^+^CD44^+^CD62L^−^ cell population representing effector memory T cells (TEMs). Analysis was conducted using a Beckman CytoFLEX flow cytometer, and the data were analyzed using FlowJo X.

### Biosafety analysis

In the unilateral tumor vaccine treatment model, the body weight and temperature of each group of mice were recorded. On day 30, the mice were euthanized. Peripheral blood was collected to assess liver function markers, including aspartate aminotransferase, alanine aminotransferase, and renal function markers, such as creatinine and blood urea nitrogen. Additionally, the hearts, livers, spleens, lungs, and kidneys of the mice were collected for histopathological evaluation. Paraffin sections of these tissues were prepared and stained with hematoxylin and eosin (H&E) to observe pathological changes.

### Statistical analysis

Statistical evaluations were conducted using the GraphPad Prism 8.0.2 software suite. Data are presented as the mean ± standard error of the mean, based on a minimum of three separate experiments. Each experimental cohort for the antitumor efficacy assessments comprised seven mice. *P*-values were determined using two-tailed unpaired Student's *t*-tests or two-way analysis of variance.

## Results

### Antitumor cytotoxicity of SKN and ICD induction in tumor cells

**Figure 2:**
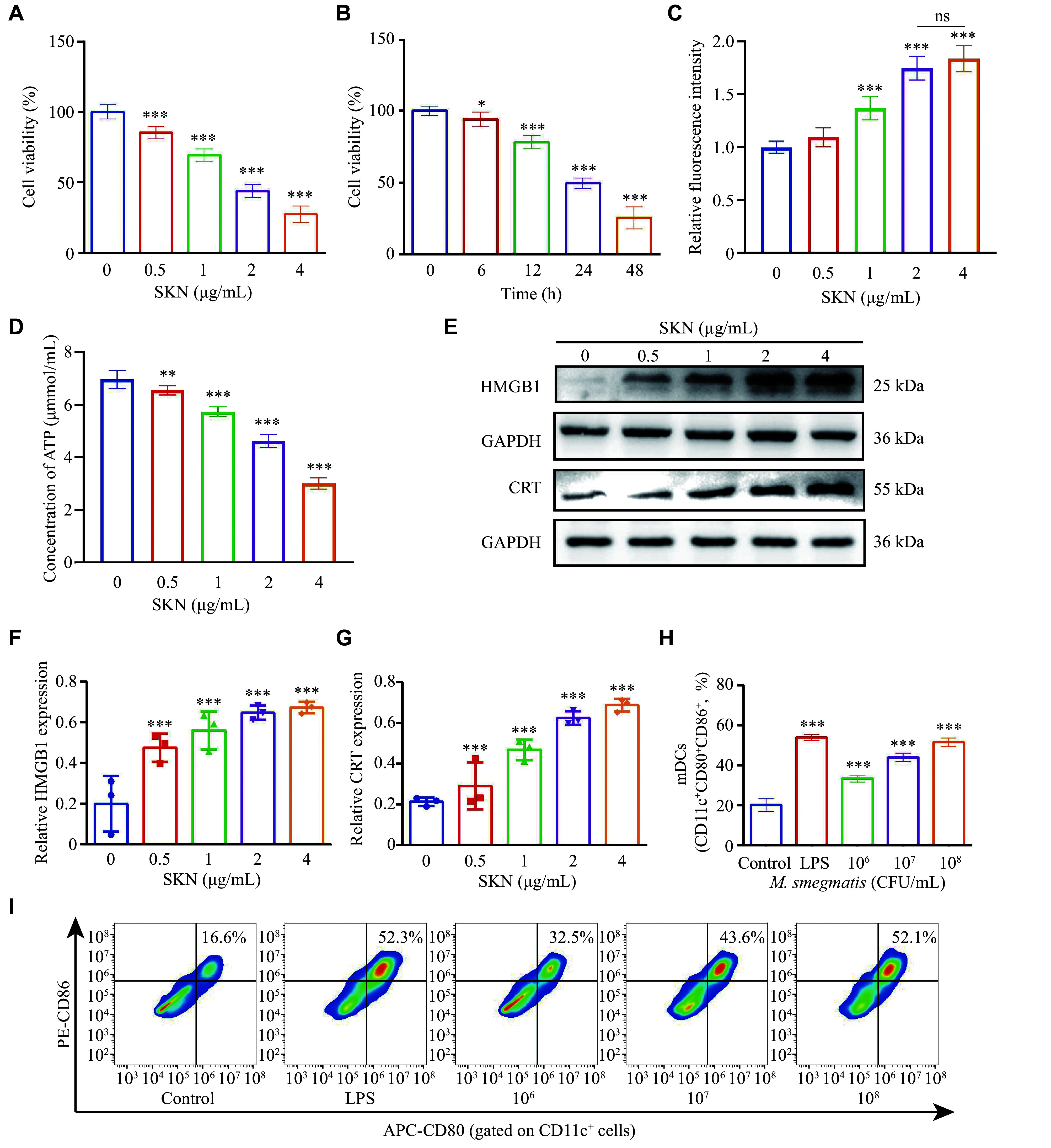
Different functions of shikonin (SKN) and *M. smegmatis* in vaccines.

To investigate the cytotoxic effect of SKN, we subjected CT26 cells, a colorectal cancer cell line, to various concentrations of SKN (0, 0.5, 1, 2, and 4 μg/mL) for 24 h. Subsequently, a CCK-8 assay was employed to assess the number of living cells and calculate cell viability. Notably, within the concentration range of 0.5 to 4 μg/mL, SKN showed a significant dose-dependent cytotoxic effect, with the calculated half-maximal inhibitory concentration (IC_50_) being 1.91 μg/mL (95% CI: 1.6 to 2.08 μg/mL), demonstrating the efficacy of SKN in inducing cell death in CT26 cells (***Fig. 2A***). Furthermore, to investigate the effects of treatment duration on the cytotoxic effect of SKN, CT26 tumor of cells were exposed to 2 μg/mL SKN for 0–48 h. The cytotoxic ability of SKN on tumor cells increased with treatment time (***Fig. 2B***). Approximately 7% of cells were killed at 6 h, which increased to approximately 50% at 24 h and 75% at 48 h.

When ICD inducers cause stress in cells, the initial response involves activation of the mechanical stress response, resulting in soluble DAMPs and cytokine release. During this process, chaperone proteins from the endoplasmic reticulum are exposed on the cell surface^[23]^. Therefore, we assessed the ROS generated during the stress response; two representative soluble DAMPs (*i.e.*, ATP and HMGB1) and the chaperone protein from the endoplasmic reticulum (*i.e.*, CRT) were used as biomarkers to identify the onset of ICD. CT26 cells were subjected to varying concentrations of SKN (0, 0.5, 1, 2, and 4 μg/mL) for 24 h, followed by measuring these indicators. A significant increase in intracellular ROS levels following SKN treatment was observed, especially at 4 μg/mL, where the relative fluorescence intensity of ROS was 1.83 times higher than that of the control group (***Fig. 2C***). Additionally, with the increase in SKN concentration, a gradual decrease in intracellular ATP content was observed, accompanied by the release of ATP outside the cell. In the 4 μg/mL SKN treatment group, the ATP content diminished by > 50%, compared with the control group (***Fig. 2D***). The results of Western blotting showed that SKN treatment significantly increased the expression levels of HMGB1 and CRT in a dose-dependent manner in CT26 cells (***Fig. 2E***–***2G***).

### *M. smegmatis* induced BMDC maturation and acted as an effective adjuvant

To confirm the effectiveness of inactivated *M. smegmatis* in activating and enhancing the function of APCs, BMDCs were isolated and cultured from the bone marrow of BALB/c mice. Various concentrations of the inactivated *M. smegmatis* were introduced to immature DCs, and flow cytometry analysis was conducted after 24 h. In DCs marked with CD11c^+^, the population of cells expressing CD80 and CD86 indicates mDCs with excellent antigen-presenting capabilities. Exposure to the inactivated *M. smegmatis* at a concentration of 1 × 10^6^ CFU/mL led to a significant increase in mDCs proportion, compared with the untreated negative control group (33.4% *vs.* 20.2%) (***Fig. 2H*** and ***2I***). An increase in the concentration of inactivated bacteria to 1 × 10^7^ CFU/mL elevated the proportion of mDCs by 43.9%, and the proportion of mDCs in the group treated with a concentration of 1 × 10^8^ CFU/mL was slightly higher (51.5%). These results indicated that the inactivated *M. smegmatis* activated DCs, which substantiates the feasibility of using inactivated *M. smegmatis* as an adjuvant in Vac-SM vaccine construction.

### Evaluation of Vac-SM antitumor effects in animal models

We initiated the intratumoral injection treatment protocol 7 days post-cell inoculation, when the tumor volume reached approximately 50 mm³. Subsequent treatments were administered on days 7, 10, 13, and 16. The mice were randomly assigned to four treatment groups: the control, *M. smegmatis*, SKN, and Vac-SM groups, each comprising seven mice. The control group was administered 100 µL of intratumoral injection of saline. The treatment groups were administered various agents, including 1 × 10^8^ CFU of *M. smegmatis*, 50 µg of SKN, and Vac-SM (50 µg of SKN mixed with 1 × 10^8^ CFU of *M. smegmatis*), all dissolved in 100 µL of saline for injection.

**Figure 3:**
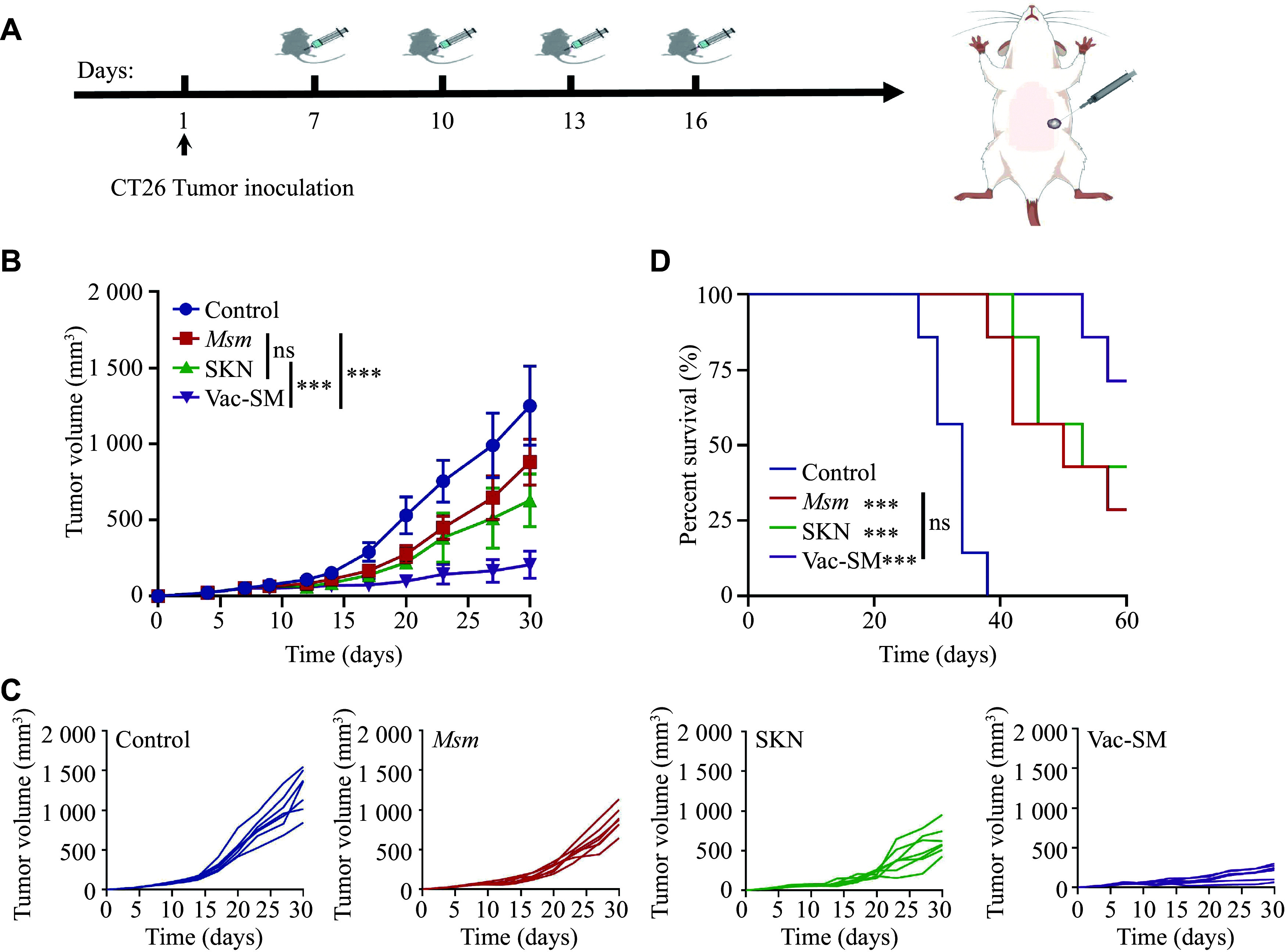
*In vivo* antitumor effect of the bifunctional *in situ* tumor vaccine Vac-SM.

The tumor volume in the control group mice increased rapidly, whereas tumor growth was effectively suppressed in the other three treatment groups (***Fig. 3B***). Specifically, Vac-SM significantly limited tumor progression, compared with that of the *M. smegmatis* and SKN groups. When examining the individual tumor growth data for all groups (***Fig. 3C***), five out of seven mice in the Vac-SM group exhibited a gradual tumor growth trend, maintaining tumor volumes below 250 mm³ after 30 days of tumor implantation, whereas the remaining two mice displayed slightly larger tumor volumes, slightly greater than 250 mm³. During the 60-day survival observation period, mice in the *M. smegmatis*, SKN, and Vac-SM treatment groups showed significantly increased survival rates, compared with those in the control group (***Fig. 3D***).

### Assessment of the distant antitumor effect of the Vac-SM vaccine in animal models

**Figure 4:**
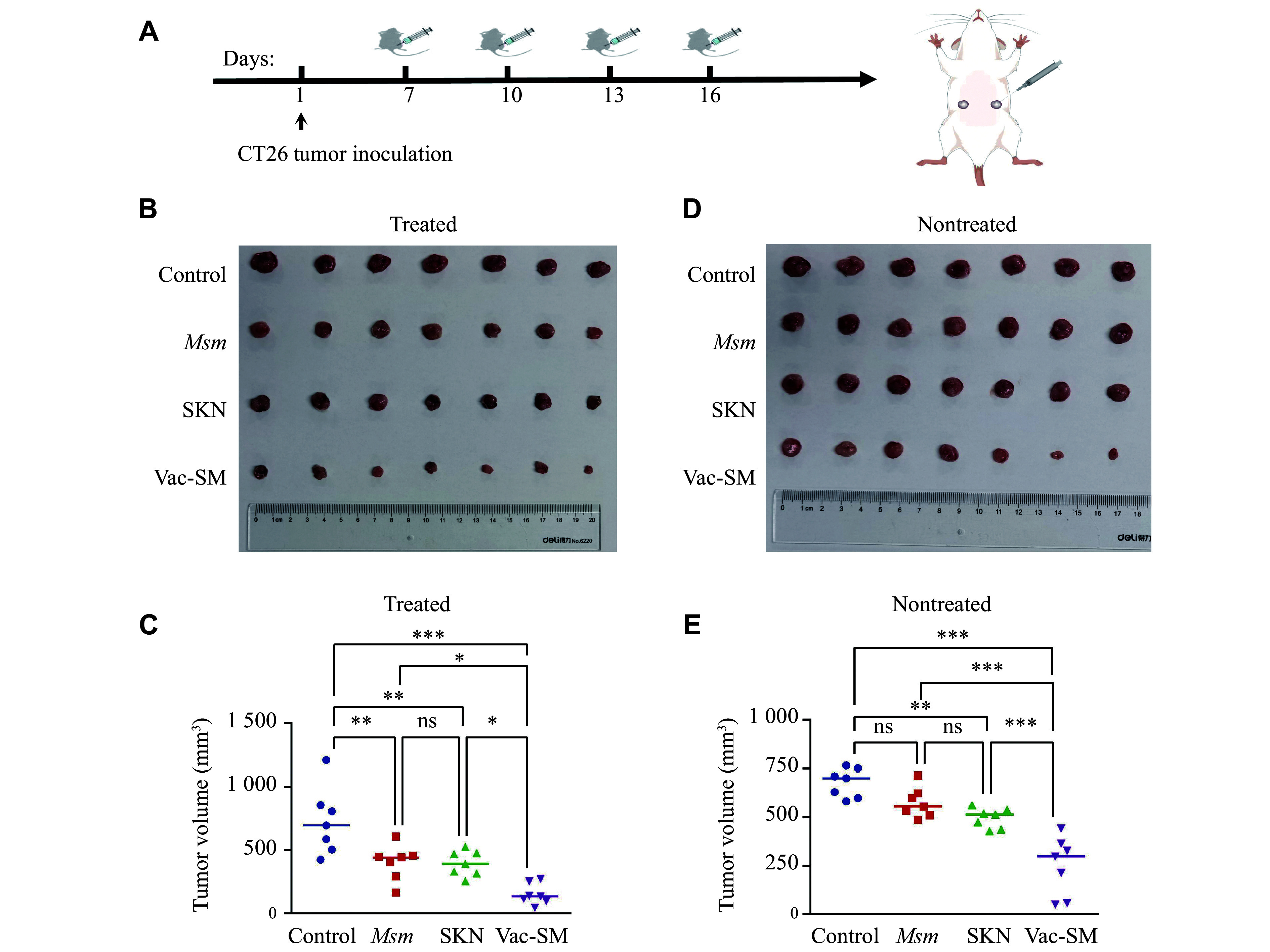
*In vivo* experiment to demonstrate the distant antitumor effect of the Vac-SM vaccine.

We inoculated 7 × 10^5^ CT26 tumor cells into both the left and right lower abdomens of BALB/c mice, thus establishing a bilateral tumor model (***Fig. 4A***). When the tumor volume increased to approximately 50 mm^3^ on day 7, we exclusively administered a local subcutaneous injection to the left tumor. The treatment groups, dosages, and frequencies remained constant with the previously described protocol^[24]^.

Compared with the control group (with an average tumor volume of 695 mm³), the *M. smegmatis* (446.7 mm³) and SKN groups (393.3 mm³) exhibited a significant decrease in growth of the treated tumors (***Fig. 4B*** and ***4C***). Particularly, the average tumor volume was reduced to 141.5 mm³ in the Vac-SM group, exhibiting the most significant inhibitory effect, compared with the control group, thereby reinforcing the potential of Vac-SM in the treatment of *in situ* tumors. Additionally, the average volume of the nontreated tumors in the SKN (513.8 mm³) and Vac-SM groups (298.5 mm³) was also significantly decreased compared with the control group, demonstrating significant distant effects, while the Vac-SM group exhibited a significantly superior effect than the SKN group (***Fig. 4B*** and ***4C***). These findings highlight the advantage of combining SKN with *M. smegmatis* in activating systemic antitumor immune responses through multiple pathways.

### Immune response induced by Vac-SM

To evaluate the immune response elicited by Vac-SM *in vivo*, we administered it subcutaneously in a bilateral tumor-bearing mouse model. After seven days from the last treatment, which was the 23rd of the experiment, all experimental mice were euthanized. We collected TDLNs (*i.e.*, inguinal lymph nodes), spleens, and tumor tissues from the treated side. Subsequently, we employed flow cytometry to analyze the immune responses in these tissues.

**Figure 5:**
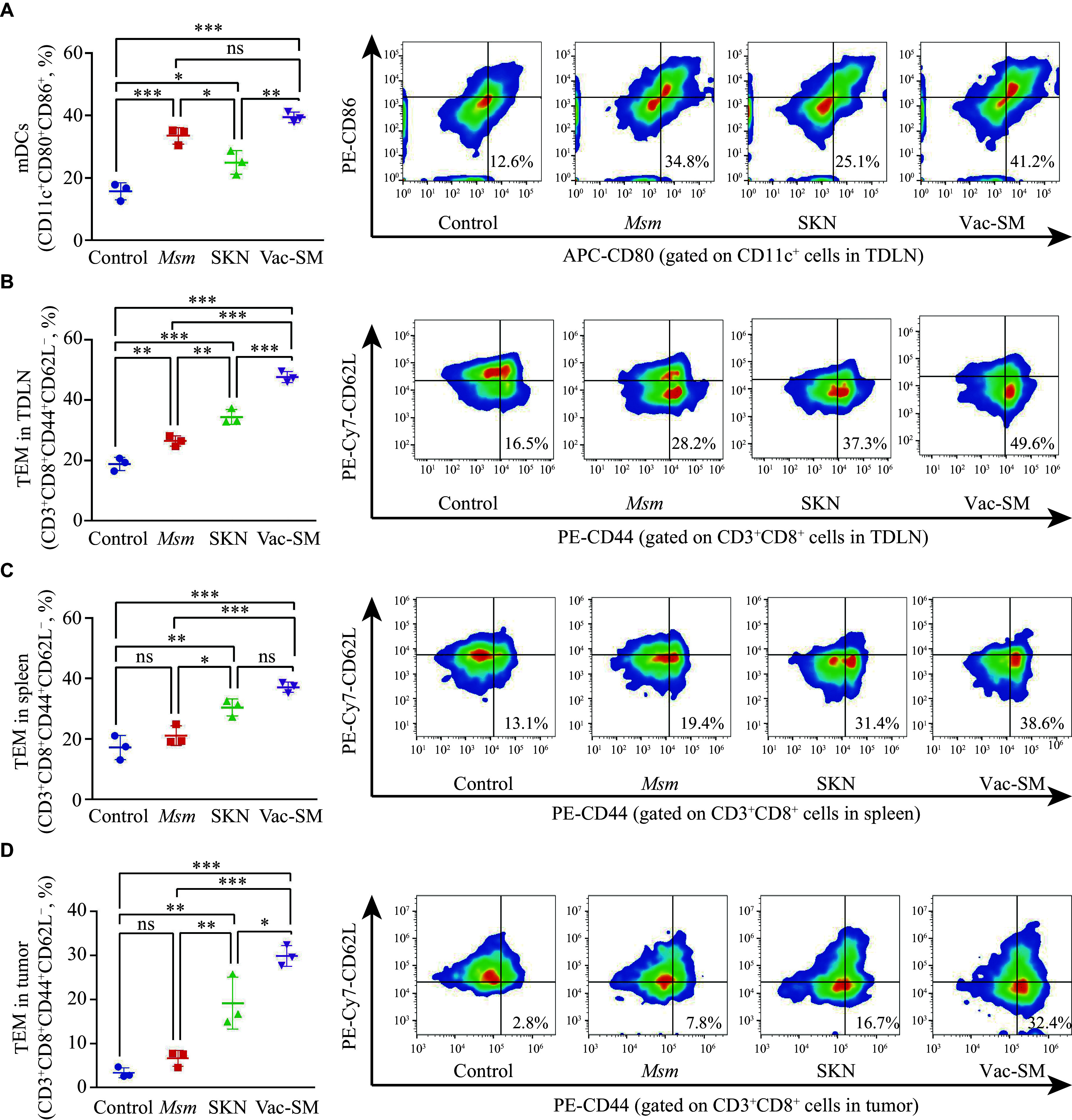
Immune responses induced by the Vac-SM vaccine.

In the TDLNs, the proportion of mDCs marked with CD11c^+^CD80^+^CD86^+^ was higher in all three treatment groups than in the control group. The two groups containing *M. smegmatis* exhibited a higher proportion of mDCs than the SKN group (***Fig. 5A***). This finding suggested that while SKN activated DCs by inducing a weak ICD, its effect was not as strong as that of *M. smegmatis*. Vac-SM combines the functions of SKN and *M. smegmatis*, which may maximize the APC activation.

TEMs (CD3^+^CD8^+^CD44^+^CD62L^−^) play an important role in secreting TNF-α and IFN-γ to eliminate tumor cells. Therefore, we examined the TEM phenotype in the TDLNs, spleen, and tumor to investigate functional T-cell activation and immune memory formation. In the TDLN (***Fig. 5B***), the average proportion of TEM distinctly formed four gradients, with the lowest observed in the control group (18.9%). This finding suggests that untreated tumor tissues face challenges in effectively releasing antigens and stimulating T-cell immunity regardless of their size. Conversely, the average TEM proportion was slightly higher at 26.5% in the *M. smegmatis* group than in the control group, indicating that the enhancement of APC function alone may help the tumor tissues present a limited number of tumor antigens, thereby activating T-cell responses to some extent. The average TEM proportion in the SKN group was 34.5%, indicating that DAMPs, tumor antigens, and cytokines released locally within the tumor tissues through ICD may activate effective T-cell immune responses. The Vac-SM group exhibited the highest average TEM proportion at 47.6%, demonstrating that supplementing an adjuvant to enhance the APC function in SKN-induced ICD activation of antitumor T-cell immunity may facilitate antigen cross-presentation, further enhancing T-cell immune activation.

The spleen is a vital immune organ essential for immune antigen stimulation and memory formation. Therefore, we also assessed TEM in the spleens of mice (***Fig. 5C***). Similar to the trend observed in the TDLNs, the average proportion of TEM was higher in all treatment groups than in the NS group (17.2%), with Vac-SM exhibiting the highest proportion (37.1%). This finding suggests that the tumor antigens released locally by the Vac-SM vaccine may establish an effective immune memory in the spleen. In the tumor tissues (***Fig. 5D***), the average TEM proportion in the *M. smegmatis* group (6.7%) increased only marginally, with no statistically significant difference from that of the control group (3.4%). However, the average TEM proportion was significantly higher in the Vac-SM group (29.9%) than in both the control group (nearly 10 times) and the SKN group (19.2%).

Combining all of the immune response results, we elucidated why Vac-SM exhibited the most effective therapeutic outcome in the mouse tumor suppression model. SKN releases antigens locally within tumors by inducing ICD. Combined with *M. smegmatis*, it enhances the function of APCs in TDLNs, activating effector T cells in TDLNs and fostering a potent immune memory in the spleen. Ultimately, this synergy enhances the infiltration of TEM into the tumor tissues, eliciting a robust systemic and specific antitumor immune response, while concurrently targeting *in situ* and metastatic (distant) tumors.

### Biosafety assessment of Vac-SM vaccines

**Figure 6:**
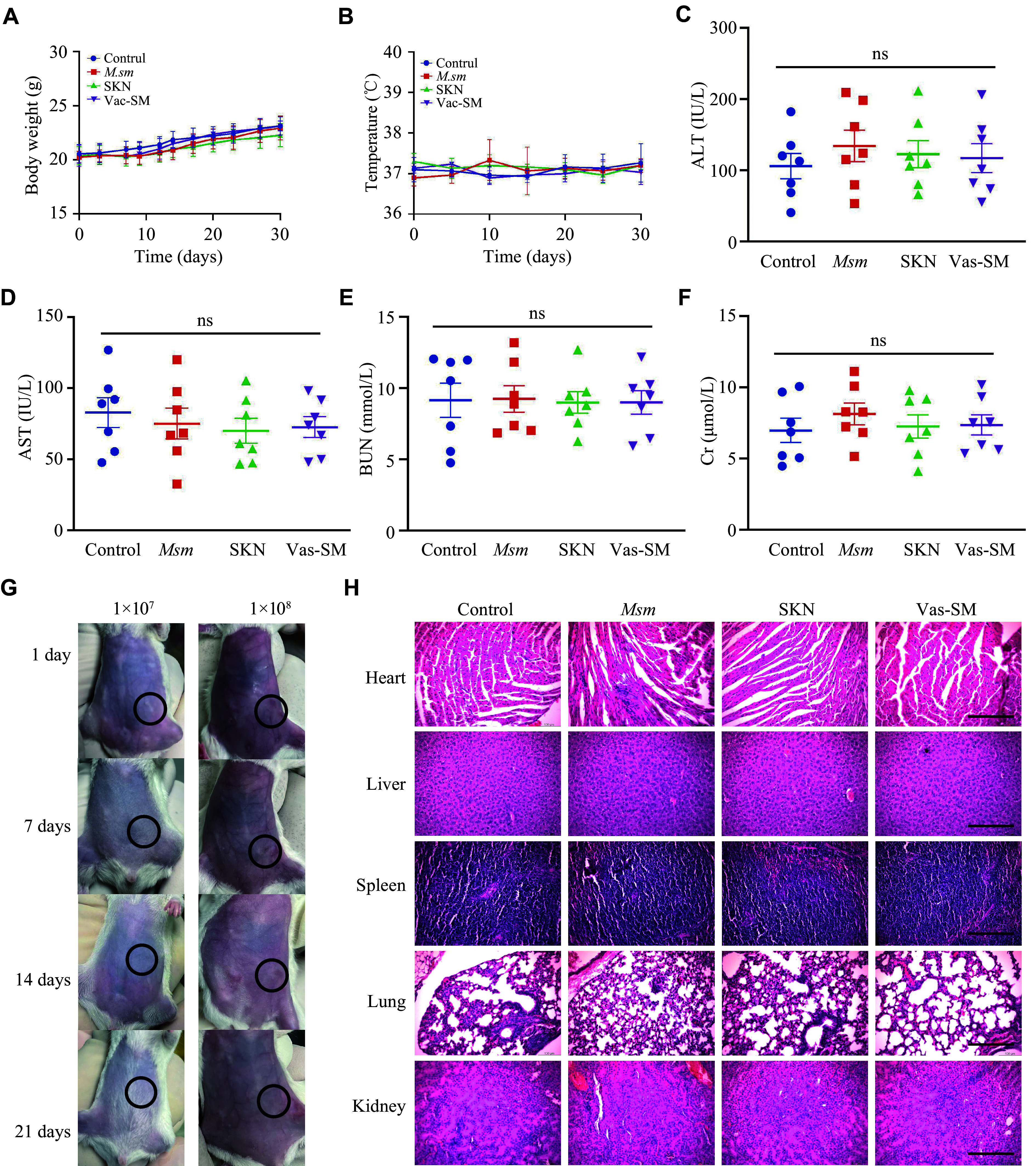
The Vac-SM vaccine showed no obvious toxicity in the mice.

To demonstrate the safety of Vac-SM vaccine, we monitored the weight, body temperature, and various organ functions of mice after injection in each group. The changes in the body weight of the mice throughout the entire treatment period showed a gradual increase with fluctuations in all groups, with no statistically significant difference observed among the treatment and control groups (***Fig. 6A***). We monitored the body temperature of the mice after subcutaneous injection of the vaccine and there was no increase in all groups (***Fig. 6B***). The changes in the liver and kidney function indicators of the mice were observed with no statistically significant differences among the treatment and control groups (***Fig. 6C***–***6F***). Moreover, H&E staining of the paraffin sections from various major organs of the mice did not reveal any pathological changes. These findings suggest that Vac-SM may be safe at the current treatment dose (***Fig. 6H***).

We further observed the degradation of *M. smegmatis* *in vivo* over time. Different concentrations of *M. smegmatis* (1 × 10^7 ^and 1 × 10^8^ CFU) were injected subcutaneously on the 1st day and were observed every few days until completely degraded. It should be noted that no obvious inflammatory responses, such as rubor, calor, swelling, and ulceration, were observed during the following time. The *M. smegmatis* was completely degraded after 20 days (***Fig. 6G***).

## Discussion

With advancements in traditional cancer treatment strategies, despite the achievement of significant success in curative treatments for primary tumors, cancer patient survival is constrained by distant metastases, resulting in death. As an emerging therapeutic modality, tumor vaccines have become a potent adjunct to traditional treatment approaches, which induce systemic antitumor immune responses and immune memory, thereby inhibiting the systemic dissemination of tumors and holding the promise for treating metastatic tumors. Hence, assessing distant antitumor immune effects is crucial for evaluating the efficacy of tumor vaccines. Personalized cancer vaccines that target specific antigens, including the MAGE-A3 peptide^[25]^ and NEO-PV-01 vaccines^[26]^, demonstrate commendable immunogenicity. However, because of the extensive heterogeneity of tumors, vaccines designed to target only one or a few tumor antigens may not elicit a comprehensive immune response. Additionally, the development and application of such vaccines face significant limitations due to the absence of universal tumor antigens. Thus, the *in situ* cancer therapies target the tumor microenvironment to enhance immunogenicity, aiming to take advantage of a more comprehensive spectrum of antigens and facilitate the generation of tumor-infiltrating lymphocytes to reverse cancer immune tolerance by inducing effective antitumor immune responses. Key strategies of the *in situ* cancer therapies encompass the intratumoral injection of immune checkpoint inhibitor antibodies, immune cells, bacteria and related formulations, nucleic acid preparations, and oncolytic viruses^[27]^.

Recently, a newly recognized form of programmed cell death, known as ICD, has emerged, which induces immunogenicity in dying cells, facilitating their recognition by the immune system^[7]^. The approach targets the existing tumor cells, stimulates the immune system for long-term surveillance, and has the ability to combat potential tumor cells, thereby preventing tumor recurrence and metastasis. The herbal extract SKN effectively inhibits the growth of various tumors and has been established as a potent inducer of ICD^[18]^. In the present study, we further demonstrated that SKN-induced ICD in CT26 cells in a dose-dependent manner by measuring levels of ROS, ATP, HMGB1, and CRT, providing a strong evidence for using SKN in producing the *in situ* tumor vaccines.

In our unilateral tumor mouse model, we observed that treatment with SKN alone was not entirely effective in suppressing tumor growth. On day 30, the average tumor volume was 629.4 mm³, only marginally better than that (882.4 mm³) observed in the *M. smegmatis* group alone. In our preliminary study, increasing the dosage and frequency of SKN did not eliminate the tumors, but led to significant side effects, such as a decrease in appetite, weight loss, and mortality in mice. These may be because of the low bioavailability of SKN, a widely recognized issue and a focal point for future investigation. Moreover, the results indicated that SKN could not sufficiently activate the DC function. Despite exposure to many tumor antigens through ICD, it struggles to effectively activate a potent T-cell immune response. Consequently, SKN treatment alone only effectively controlled tumor growth in the early stages; however, in the later stages of treatment or after treatment cessation, maintaining control over tumor growth becomes challenging. This is consistent with the findings from our animal model vaccine treatment experiments. To enhance the therapeutic efficacy of the SKN vaccine, we hypothesized that the insufficient activation of APC functions might be a factor. Therefore, we supplemented the SKN vaccine with the adjuvant *M. smegmatis* to enhance DC activation.

In a preliminary study performed in our laboratory, we designed a hybrid membrane hydrogel tumor vaccine using *Mycobacterium phlei* and tumor cell membranes^[28]^. We used *M. smegmatis* as an adjuvant to activate DCs to enhance the antitumor immune response triggered by the SKN vaccine. However, to avoid potential infections associated with live bacteria and simplify vaccine preparation, we prefer using the inactivated *M. smegmatis* instead of *M. smegmatis* membranes. The most favorable therapeutic outcome was achieved by combining SKN with *M. smegmatis* to construct Vac-SM. Correspondingly, the Vac-SM significantly activated DCs in TDLNs, promoting the transformation of T cells to TEMs in the TDLN. An increase in TEMs within the tumor tissues suggested that the Vac-SM effectively activated antitumor T-cell immunity, leading to the successful infiltration of effector T-cells into the tumor tissues. Additionally, the increased proportion of TEMs in the spleen indicated that the Vac-SM vaccine established an effective immune memory, thereby endowing the Vac-SM with the most potent distant antitumor effects. Therefore, the Vac-SM represents an effective, reliable, and safe *in* *situ* tumor vaccine with a promising clinical translation potential.

Although the Vac-SM effectively suppresses tumor growth by triggering a robust T-cell immune response, it is inadequate for complete tumor eradication in the long term and may have limited efficacy against distant metastatic tumors. These challenges are widespread in many therapeutic vaccines, characterized by Treg overactivation and T-cell exhaustion^[29–30]^. Notably, the release of excessive tumor antigens triggers immune checkpoints. In one study, after vaccine treatment, both *in situ* and distant tumors showed a significant increase in PD-L1 expression, accompanied by the elevated PD-1 expression in T cells, elucidating the gradual diminishment of the therapeutic effects of vaccines over time^[28]^. In another study using pingyangmycin as an ICD inducer combined with PD-1 antibodies, PD-1 antibodies effectively enhanced the ICD-induced T-cell immunity, such as an increase in tumor-infiltrating CD8^+^ T cells, a decrease in the proportion of CD4^+^ T cells, and a reduction in the proportion of Tregs in the peripheral blood^[31]^. Hence, integrating the Vac-SM with immune checkpoint inhibitors may result in a highly effective combined immunotherapy regimen.

In conclusion, we developed an in-situ tumor vaccine Vac-SM, based on SKN and a bacterial adjuvant *M. smegmatis*, with the significant distal anti-tumor efficacy and safety, showing a promising potential for clinical translation.
